# Impact of TLR7 Genetic Variants rs179008 and rs3853839 on Susceptibility to Severe Coronavirus Disease 2019

**DOI:** 10.1002/iid3.70585

**Published:** 2026-11-13

**Authors:** Maryam Noroozi, Gholamreza Daryabor, Reza Shafiei, Mona Fani, Seppo Meri, Kurosh Kalantar

**Affiliations:** ^1^ Department of Immunology, School of Medicine Shiraz University of Medical Sciences Shiraz Iran; ^2^ Autoimmune Diseases Research Center Shiraz University of Medical Sciences Shiraz Iran; ^3^ Vector‐Borne Diseases Research Center North Khorasan University of Medical Sciences Bojnurd Iran; ^4^ Helsinki University Hospital, The University of Helsinki and HUSLAB Helsinki Finland

**Keywords:** coronavirus disease 2019 (COVID‐19), severe acute respiratory syndrome coronavirus 2 (SARS‐CoV‐2), single‐nucleotide polymorphisms (SNPs), Toll‐like receptor 7 (TLR7)

## Abstract

**Background:**

Coronavirus disease 2019 (COVID‐19) exhibits a broad clinical spectrum, ranging from asymptomatic infection to severe pneumonia and respiratory failure. Accumulating evidence indicates that host genetic variations, particularly those governing innate immune responses and type I interferon (IFN‑I) production, critically dictate disease trajectories. Toll‐like receptor 7 (TLR7), an X‐linked sensor that recognizes single‐stranded RNA viruses, is a fundamental initiator of antiviral IFN‐I responses. Hence, functional polymorphisms within the TLR7 gene may substantially alter individual susceptibility to severe COVID‐19.

**Objective:**

This hospital‐based case‐control study aimed to evaluate the association of two *TLR7* single‐nucleotide polymorphisms (SNPs), rs179008 and rs3853839, with COVID‐19 severity, with particular attention to sex‐specific effects given the X‐chromosomal location of the gene.

**Methods:**

A total of 377 confirmed COVID‐19 patients, stratified into severe (*n* = 182) and mild (*n* = 195) groups, were matched for age and sex. Genotyping was performed using polymerase chain reaction–restriction fragment length polymorphism (PCR‐RFLP). All statistical analyses were rigorously stratified by sex to account for the X‐linked inheritance pattern of TLR7.

**Results:**

For rs179008, the T allele exhibited a protective trend in both females (OR = 0.57, *p* = 0.085) and males (OR = 0.61, *p* = 0.213). Importantly, the TT genotype was absent in severe female cases but present in 8.2% of mild female cases, indicating a significant protective effect (*p* = 0.029). Notably, pooling both sexes revealed a significant overall protective association for the T allele (overall frequency 13.4% in severe vs. 20.8% in mild; crude OR = 0.59, 95% CI: 0.37–0.95, *p* = 0.029). In the case of rs3853839, a strong association was observed in females; the heterozygous CG genotype was associated with a 2.2‐fold increased risk of severe disease compared to CC homozygotes (OR = 2.20, 95% CI: 1.13–4.29, *p* = 0.019), while a recessive model supported a protective effect of the CC genotype (OR = 0.50, 95% CI: 0.26–0.94, *p* = 0.032). In the combined allele analysis across both sexes, the G allele was significantly overrepresented in severe cases (30.8% vs. 23.0%; crude OR = 1.49, 95% CI: 1.01–2.19, *p* = 0.043), confirming a global risk effect consistent with the sex‑specific trends.

**Conclusions:**

Our findings demonstrate that *TLR7* polymorphisms rs179008 and rs3853839 are significantly associated with COVID‐19 severity, with distinct sex‐specific effects. The T allele of rs179008 confers protection, while the G allele of rs3853839 increases disease risk. These results underscore the importance of sex‐stratified genetic analyses and suggest that *TLR7* variants may serve as potential biomarkers for risk stratification in COVID‐19 patients.

## 1 Introduction

In early December 2019, an outbreak of Coronavirus disease 2019 (COVID‐19), caused by the severe acute respiratory syndrome coronavirus 2 (SARS‐CoV‐2), occurred in Wuhan, Hubei Province, which was declared a pandemic by the World Health Organization in March 2020 [1, 2]. Most people infected with SARS‐CoV‐2 will experience mild to moderate respiratory illness and recover without special treatment [3]. However, a subset of patients develops severe pneumonia, acute respiratory distress syndrome (ARDS), and multi‐organ failure, requiring intensive care support [4, 5]. Typical clinical symptoms of COVID‐19 range from mild to severe respiratory illness that may lead to long‐term reduction in lung function and death [6]. The mortality rate among severe COVID‐19 patients has been reported to range between 1% and 5%, depending on the population and healthcare capacity [7]. Once the virus gains access to the body, the host's immune system recognizes its single‐stranded RNA genome through engagement of pathogen recognition receptors (PRRs). The RNA‐sensing PRRs, retinoic acid‐inducible gene I (RIG‐I) and toll‐like receptor 7 (TLR‐7), are involved in immunity against RNA viruses through induction of type I interferons (IFN‐I) production [8, 9]. Given that SARS‐CoV‐2 has been shown to inhibit RIG‐I signaling, the role of TLR7 in antiviral immunity may become particularly critical [10, 11]. TLR7 is an endosomal pattern‐recognition receptor that detects single‐stranded RNA viruses, such as SARS‐CoV‐2, and triggers the production of type I interferons [12, 13]. This receptor plays a crucial role in activating the Nuclear factor‐kappa B (NF‐κB) pathway, producing cytokines, and conferring resistance to pathogens [3]. For instance, activation of TLR7 on monocytes, macrophages, and dendritic cells (DCs) leads to the production of interleukin‐1 (IL‐1), interleukin 6 (IL‐6), monocyte chemoattractant protein 1 (MCP‐1), α‐tumor necrosis factor (α‐TNF), and Type I interferons (IFN‐I) [14]. IFN‐I is one of the key inhibitors against SARS‐CoV‐2 infection [15]. However, IFN‐I, as an inflammatory factor, may cause severe symptoms in COVID‐19 patients [16]. Evidence has shown that the function of IFN‐I is time‐dependent [17, 18]. An initial peak of IFN‐I has been observed in patients with mild to moderate COVID‐19 [19, 20]. A strong and well‐timed IFN response is considered protective. However, if this response is uncontrolled and untimely, increased cytokines and chemokines, many of which are IFN‐stimulated genes (ISGs), can lead to acute respiratory distress syndrome (ARDS) and other severe complications of this disease [16, 21]. Hence, both early deficits in type I IFN responses and late IFN hyper‐responsiveness are hallmarks of severe COVID‐19. Single‐nucleotide polymorphisms (SNPs) may profoundly alter immune responses to SARS‐CoV‐2, potentially serving as critical determinants of disease outcomes [22, 23]. The TLR7 gene is located on the X chromosome (Xp22.2) and contains several functional polymorphisms that may modulate immune responses. Thus, we hypothesized that TLR7 polymorphisms may affect the susceptibility and clinical outcomes of SARS‐CoV‐2 infection. Among these, rs179008 is a missense variant located in exon 3 of TLR7 (chromosome X, position 12,885,540 on GRCh38; c.32 A > T), which has been implicated in altering TLR7 expression and function. The importance of rs179008 in defense against hepatitis C virus (HCV) [24], human immunodeficiency virus (HIV) [25], and dengue virus [26] has been well indicated. Another TLR7 genetic variant, rs3853839, is located in the 3′ untranslated region (3′ UTR) of the gene (chromosome X, position 12,889,539 on GRCh38) and has been shown to affect TLR7 mRNA stability by modulating microRNA binding [27]. This SNP is also implicated in the pathogenesis of hand, foot, and mouth disease (HFMD) caused by Enterovirus 71 (EV71) [28]. Accordingly, this study was conducted to investigate the association of rs179008 and rs3853839 polymorphisms in the TLR7 gene on the severity of COVID‐19. Given the X‑chromosomal location of TLR7, sex‑stratified analyses are essential to fully understand the impact of these variants, as females may exhibit mosaic expression and gene dosage effects due to the X‑chromosome inactivation process, while males are hemizygous.

## 2 Materials and Methods

### 2.1 Study Population

This investigation was conducted as a hospital‐based case–control study involving 377 patients with confirmed COVID‐19. The study population consisted of 182 patients with severe disease who were classified as the case group and 195 patients with mild disease as the control group. Nasopharyngeal swabs or lower respiratory tract samples were collected between June and August 2021 from confirmed COVID‐19 patients. All subjects, both in the severe and mild groups, were confirmed to have SARS‐CoV‐2 by polymerase chain reaction (PCR). Mild patients presented with fever, cough, sore throat, fatigue, headache, muscle pain, nausea, vomiting, diarrhea, and loss of sense of taste or smell. With respect to the severe group, patients were included because they required hospitalization in the intensive care unit (ICU) or the use of a ventilator. Patients with common underlying diseases (including hypertension, cardiovascular disease, diabetes, chronic liver disease, autoimmune diseases, and cancer) and those who later died from COVID‐19 were excluded from both groups. Individuals who were biologically related to another enrolled participant (first‐ or second‐degree relatives) were excluded from the study to avoid potential bias due to shared genetic background [29].

### 2.2 Genomic DNA Extraction

Genomic DNA was extracted from 2 mL of anticoagulated (EDTA) peripheral venous blood from all patients by the salting‐out method [30, 31, 32, 33]. The DNA concentration and its purity were determined by measuring absorbance at 260 and 280 nm wavelengths. The DNA extracts were stored at −20°C until use.

### 2.3 Analysis of the Genotypes and Alleles of rs179008 and rs3853839

The genetic variations of TLR7 rs179008 and rs3853839 were determined by polymerase chain reaction–restriction fragment length polymorphism (PCR‐RFLP). The polymorphic sequence‐containing regions were amplified by PCR in a total volume of 10 μL containing 5 μL of 2× Taq PreMix Blue (Parstous, Iran, Mashhad), 0.3 μL of each primer (Table 1) [34, 35] (CinnaGen, Iran, Tehran), 1 μL DNA containing 30 ng genomic DNA, and 3.4 μL H_2_O. The PCR conditions for all SNPs were set as follows: initial denaturation at 95°C for 5 min, followed by 30 cycles of denaturation at 95°C for 30 s, annealing at different temperatures (61°C for rs179008 and 63°C for rs3853839) for 30 s, and extension at 72°C for 30 s, with a final extension at 72°C for 5 min. The PCR product sizes (rs179008, 369 bp; rs3853839, 277 bp) were visualized on a 3% agarose gel stained with Safe Stain dye (CinnaGen, Iran, Tehran) to assess amplification quality. Then, 5 μL of PCR product was incubated at 37°C for 16 h with the appropriate restriction enzyme (Thermo Fisher Scientific, Waltham, MA, USA; Table 1).

**Table 1:** The primer sequence, restriction enzymes, and fragment length of the rs179008 and rs3853839.

SNP	Location	Primers (5′‐3′)	Ta (◦C)	Enzyme	Genotypes and fragments (bp)
rs179008	Exon 3	F: 5′ TAACAACGAATAGGAAAATGC3′ R: 5′ GTTTTAGGAAACCATCTAGCC3′	61	ApoI	TT:211 + 158 AT: 211 + 158 + 92 + 66 AA: 211 + 92 + 66
rs3853839	3′ UTR	F: 5′ TTGCTTCCGTGTCATCCAGG3′ R: 5′ ACAGTACTTTGCAGTGCAGATAAA3′	63	StyI	CC: 185 + 92 CG: 277 + 185 + 92 GG: 277

Abbreviations: SNP, single‐nucleotide polymorphism; Ta, annealing temperature.

### 2.4 Statistical Analysis

The Statistical Package for Social Sciences (SPSS) software (version 26) and EPI INFO software were used for statistical analyses. Genotype and allele frequencies between severe and mild patients were compared using the *χ* ^2^ test. For comparisons with small, expected cell counts (≤ 5), Fisher's exact test (two‑sided) was applied instead. Odds ratios (ORs) and their 95% confidence intervals (CIs) were calculated to estimate the strength of the association between each genotype/allele and disease severity. Given the X‑chromosomal location of TLR7, all analyses were meticulously stratified by sex to account for the hemizygous state in males and the homozygous/heterozygous states in females. All genotype frequencies were tested for deviation from Hardy–Weinberg equilibrium using the *χ* ^2^ test, stratified by sex due to TLR7 location on the X‑chromosome. Both SNPs were found to be in equilibrium (*p* > 0.05) for both SNPs in the overall cohort and within each sex (tested on allele frequencies for hemizygous males). A *p* value < 0.05 was considered statistically significant for all comparisons.

## 3 Results

### 3.1 Representative PCR–RFLP Electrophoretic Patterns of TLR7 Polymorphisms

The restricted fragments were separated by electrophoresis on a 3% agarose gel (Invitrogen, Thermo Fisher Scientific, Waltham, MA, USA) containing Safe Stain and visualized using an ultraviolet transilluminator (Accuris UV Transilluminators, Tucson, USA; Figure 1).

**Figure 1:**
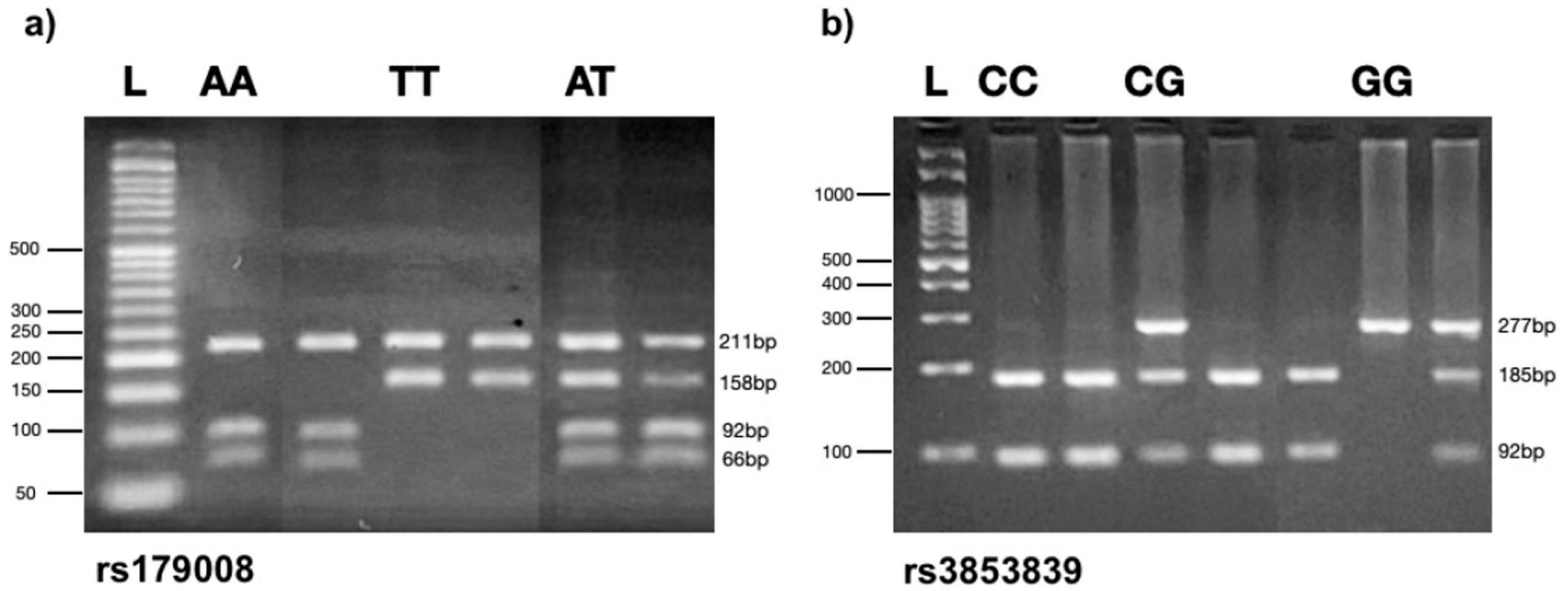
Representative PCR‑RFLP electrophoretic patterns of TLR7 rs179008 and rs3853839 resolved on a 3% agarose gel. (a) rs179008 digestion products: the TT genotype yields two fragments (211 and 158 bp); AT yields four fragments (211, 158, 92, and 66 bp); and AA yields three fragments (211, 92, and 66 bp). (b) rs3853839 digestion products: CC yields two fragments (185 and 92 bp); CG yields three fragments (277, 185, and 92 bp); and GG yields a single undigested fragment (277 bp).

### 3.2 Patient Cohort and Baseline Characteristics

Initially, 844 patients were evaluated. Following the application of exclusion criteria, specifically incomplete clinical or laboratory records, missing genotyping data, and pre‐existing severe comorbidities, a total of 377 patients were included in the final analysis. These were categorized into mild (*n* = 195) and severe (*n* = 182) groups. The mild group consisted of 79 females (40.5%) and 116 males (59.5%), whereas the severe group included 78 females (42.9%) and 104 males (57.1%). The mean age was 40.5 ± 9.8 years in the mild group and 40.4 ± 9.2 years in the severe group, indicating effective age matching. The study protocol received ethical approval (IR.SUMS.SEC.1401.569), and all participants provided written informed consent.

### 3.3 Genotype and Allelic Distributions of rs179008 and rs3853839

Genotyping for rs179008 was successful in 356 of the 377 patients (severe: *n *= 174, 72 females and 102 males; mild: *n *= 182, 73 females and 109 males). The remaining 21 samples failed post‐PCR quality control thresholds (e.g., low signal intensity or ambiguous clustering) specifically for this assay. Notably, the genotype failure rate did not significantly differ between the severe (8/182, 4.4%) and mild (13/195, 6.7%) groups, confirming that missingness occurred independently of disease severity. In contrast, the rs3853839 assay achieved a 100% genotyping call rate and was therefore completed in all 377 patients (severe: *n *= 182, 78 females and 104 males; mild: *n *= 195, 79 females and 116 males). Detailed association results are provided in Table 2.

**Table 2:** Association of *TLR7* SNPs (rs179008 and rs3853839) with severe COVID‐19, stratified by sex.

SNP	Sex	Genotype/allele	Severe (*n*, %)	Mild (*n*, %)	Total (*n*, %)	OR (95% CI)	*p* value
rs179008	Female	AA	52 (72.2)	47 (64.4)	99 (68.3)	1.00 (ref)	—
		AT	20 (27.8)	20 (27.4)	40 (27.6)	0.90 (0.43–1.89)	0.850
		TT	0 (0.0)	6 (8.2)	6 (4.1)	0.00^a^	0.029*
		*Dom (AA vs. AT* + *TT)*	52 vs. 20	47 vs. 26	99 vs. 46	1.44 (0.71–2.91)	0.302
		*Rec (TT vs. AA* + *AT)*	0 vs. 72	6 vs. 67	6 vs. 139	0.00^a^	0.029*
	Female	Allele A	124 (86.1)	114 (78.1)	238 (82.1)	1.00 (ref)	—
		Allele T	20 (13.9)	32 (21.9)	52 (17.9)	0.57 (0.31–1.06)	0.085
	Male	Allele A	89 (87.3)	88 (80.7)	177 (83.9)	1.00 (ref)	—
		Allele T	13 (12.7)	21 (19.3)	34 (16.1)	0.61 (0.29–1.30)	0.213
	Combined (M + F)	Allele A	213 (86.6)	202 (79.2)	415 (82.8)	1.00 (ref)	—
		Allele T	33 (13.4)	53 (20.8)	86 (17.2)	0.59 (0.37–0.95)*	0.029*
rs3853839	Female	CC	34 (43.6)	48 (60.8)	82 (52.2)	1.00 (ref)	—
		CG	39 (50.0)	25 (31.6)	64 (40.8)	2.20 (1.13–4.29)	0.019*
		GG	5 (6.4)	6 (7.6)	11 (7.0)	1.18 (0.33–4.17)	0.768
		*Dom (GG vs. CC* + *CG)*	5 vs. 73	6 vs. 73	11 vs. 146	0.83 (0.24–2.85)	0.768
		*Rec (CC vs. CG* + *GG)*	34 vs. 44	48 vs. 31	82 vs. 75	0.50 (0.26–0.94)	0.032
	Female	Allele C	107 (68.6)	121 (76.6)	228 (72.6)	1.00 (ref)	—
		Allele G	49 (31.4)	37 (23.4)	86 (27.4)	1.50 (0.91–2.47)	0.109
	Male	Allele C	73 (70.2)	90 (77.6)	163 (74.1)	1.00 (ref)	—
		Allele G	31 (29.8)	26 (22.4)	57 (25.9)	1.47 (0.80–2.69)	0.213
	Combined (M + F)	Allele C	180 (69.2)	211 (77.0)	391 (73.2)	1.00 (ref)	—
		Allele G	80 (30.8)	63 (23.0)	143 (26.8)	1.49 (1.01–2.19)*	0.043*

Abbreviations: Dom, dominant; Res, recessive.

^a^ OR of 0.00 arises from zero severe cases in that genotype group.

*Asterisked OR and *p* value are crude estimates (unadjusted for sex), calculated from the combined 2 × 2 table, and are shown for completeness alongside the sex‐stratified results.

For rs179008, the TT genotype was not observed in severe female cases (0/72), while it was present in 8.2% of mild female cases (6/73), suggesting a possible protective effect (*p* = 0.029, Fisher's exact test). However, the odds ratio could not be defined due to the zero count. The T allele showed a trend toward protection in females (OR = 0.57, 95% CI: 0.31–1.06, *p* = 0.085) and a similar but non‐significant trend in males (OR = 0.61, 95% CI: 0.29–1.30, *p* = 0.213). When sexes were pooled, the overall T allele frequency was significantly lower in severe cases (13.4%) than in mild cases (20.8%), yielding a crude OR of 0.59 (95% CI: 0.37–0.95, *p* = 0.029). No further statistically significant associations were identified for this SNP.

By contrast, rs3853839 demonstrated significant sex‐specific effects primarily in females. Among females, the heterozygous CG genotype was associated with a 2.2‐fold increase in the risk of severe disease compared with the CC genotype (OR = 2.20, 95% CI: 1.13–4.29, *p* = 0.019). The recessive model (CC vs. CG + GG) supported a protective effect of the CC genotype (OR = 0.50, 95% CI: 0.26–0.94, *p* = 0.032). In males, although the G allele was more frequent in severe cases (29.8%) than in mild cases (22.4%), this difference was not statistically significant (OR = 1.47, 95% CI: 0.80–2.69, *p* = 0.213). Notably, pooling both sexes demonstrated that the G allele was significantly overrepresented in the severe group (30.8% vs. 23.0% in mild), with a crude OR of 1.49 (95% CI: 1.01–2.19, *p* = 0.043). Thus, the combined allele analysis confirms an overall risk effect of the G allele that aligns with the direction observed in females and males but reaches significance only when both sexes are considered together.

## 4 Discussion

Early in infection, epithelial cells drive type I IFN production through RIG‐1, with minimal TLR7 expression. Thus, TLR7 SNPs likely have a greater impact in later stages, when immune cell recruitment and activation require regulation of IFN‐I to prevent excessive inflammation [36, 37]. The lower expression of TLR7 at this point can be fundamental for the best function of the immune system. The genetic variations of TLR7 affect an individual's susceptibility or resistance to viral infections. TLR7 activation is necessary for Type‐I IFN production. SNPs in the TLR7 gene may alter the production of IFN‐I, which could affect the pathophysiology of COVID‐19 [38]. In our study, carriers of the TT genotype of TLR7 rs179008 were more likely to have mild COVID‐19, with the T allele showing a protective trend, while the A allele trended toward increased risk. Notably, the TT genotype was absent among severe female cases (0/72) compared to 8.2% (6/73) in mild female cases. Similarly, previous research reported that the AA genotype was an independent risk factor for severe COVID‐19, and the AT genotype was more frequent in mild to moderate cases, suggesting a potential protective role [39]. In addition, Azar et al. reported that IFN‐I production was reduced in Plasmacytoid dendritic cells (pDCs) from A/T carrier women with mild HIV disease compared with A/A, indicating the importance of this SNP in IFN‐I production and disease outcomes. Overall, they conclude that there is a beneficial association between the T allele and lower IFN‐I production, which leads to a milder form of HIV disease [40]. The A allele leads to higher production of IFN‐I [41], resulting in greater activation and recruitment of immune cells, leading to excessive inflammation. The T allele leads to decreases in the expression of TLR7, and as a result, decreases in IFN‐I and, subsequently, interferon‐stimulated genes (ISGs) [25, 40, 42, 43]. The T allele of this SNP may, therefore, protect us against the coronavirus. This protective direction is further supported by our pooled allele analysis, which showed a significant overall reduction in T allele frequency among severe cases (OR = 0.59, *p* = 0.029), confirming that the effect is not merely a female‑specific phenomenon but is consistent across sexes when statistical power is increased.

In contrast with our results, a case‐control study including 136 patients with COVID‐19 pneumonia and 100 control groups showed that the “T/T” genotype and the T allele were associated with a significant increase in the risk of suffering COVID‐19 pneumonia [44]. Another study showed that the mutated TT genotype and the T allele at rs179008 were significantly associated with an increased risk of severe COVID‐19 and disease outcomes, reporting that men with the T/− genotype were more susceptible to COVID‐19 pneumonia than women with the same genotype [45].

The discrepancy may reflect differences in the study design and population characteristics. Unlike prior reports, we excluded major confounders (hypertension, diabetes, and chronic liver disease) and included a larger sample size. Viral genetic variability, exposure duration, and ethnic differences may also contribute. Additionally, population‐specific polymorphisms in IFN‐1–related genes such as RIG‐1 could influence IFN‐1 production and disease severity, potentially explaining the divergent findings [46]. As TLR7 is located on the X chromosome, sex‐specific analysis is essential. In our cohort, the TT genotype was protective against severe disease in women; however, the allelic association did not reach statistical significance, possibly due to limited sample size. In males, the T allele showed a similar protective trend but was also non‐significant. Although one additional study reported the T allele as a COVID‐19 risk factor, most of their cohort were men, and no sex‐stratified analysis was conducted [47]. As mentioned, in both men and women with the TT genotype, TLR7 is generally produced in lower amounts than in individuals with the AA genotype. However, in women, approximately 30% of immune cells present this gene on both X chromosomes, which is called bi‐allelic expression [48, 49]. Therefore, in women with the TT genotype, IFN‐I is neither too much nor too little. In other words, in women with the TT genotype, IFN‐I is balanced, while men with this genotype may suffer from interferon deficiency. A case report study stated that SARS‐CoV‐2 caused hepatitis in a male child carrying the T allele, suggesting that hepatitis by SARS‐CoV‐2 infection could be associated with ineffective innate immune responses caused by this polymorphism [50]. These findings suggest that the T allele may be protective in women but could contribute to IFN‐I deficiency and severe disease in men. This hypothesis is supported by a study by Eero Lauhkonen et al., which reported lung dysfunction in girls with a history of bronchiolitis who carried the A allele compared with non‐carriers [51]. This study supports our hypothesis that the AA genotype may enhance immune activation and disease severity, whereas the TT genotype is associated with milder disease, potentially through better regulation of interferon responses in women.

The most compelling finding of our study concerns the second *TLR7* SNP, rs3853839, located in the 3′ UTR. Our genotypic analysis revealed a strong and significant association in females: the CG genotype was associated with a 2.2‐fold increased risk of severe COVID‐19 compared to CC homozygotes. Consistently, the recessive model (CC vs. CG + GG) supported a protective role for the CC genotype. In males, despite a modest overrepresentation of the G allele in severe cases (29.8%) compared to mild cases (22.4%), this difference did not reach statistical significance. Importantly, the combined allele analysis (pooling both sexes) yielded a significant crude OR of 1.49 (*p* = 0.043) for the G allele, mirroring the effect sizes observed in females (OR = 1.50) and males (OR = 1.47) individually. This consistency across strata, together with the significant pooled result, strongly suggests that the G allele is genuinely associated with increased severity and that the lack of significance in male and female allele‑wise tests was largely due to reduced subgroup sample sizes. Hence, the C allele of rs3853839 in our population appears to be predominantly protective, while the G allele significantly increases the risk of severe disease.

This hypothesis is supported by Shen et al., who identified the G allele as a risk factor for systemic lupus erythematosus and demonstrated that rs3853839 was a functional polymorphism influencing IFN‐I production. They reported higher TLR7 transcript levels and enhanced IFN‐I expression in G allele carriers, with heterozygous individuals exhibiting 2.7‐fold greater G than C allele expression [52]. Two other studies have also obtained the same results and reported that the rs3853839 variant was associated with an increase in SLE risk [53, 54]. Following the above studies, a study that has examined the performance of rs3853839 shows that allele G and GG genotypes increase TLR7 expression, which is significantly associated with COVID‐19 exacerbation. They found that the GG genotype is more common among patients with critical illness and is associated with severe clinical complications [55]. Another study in Mexico reached a similar result and stated that the GG genotype of rs3853839 was associated with the critical outcome of COVID‐19 [56]. Given that TLR7 is X‐linked, we performed a sex‐stratified analysis. In women, the CC genotype was associated with mild COVID‐19, while GC carriers developed severe disease, suggesting a protective effect of CC. No significant allelic differences were observed among women with mild versus severe disease. In men, neither alleles nor genotypes differed between severity groups. Similarly, Aya Kawasaki et al. reported that the G allele and GG genotype of this SNP were significantly associated with SLE susceptibility in Japanese women [57]. Also, Raafat and colleagues showed the possible role of rs3853839 in the pathogenesis of the Egyptian female population suffering from SLE [58]. Rs3853839 is a single nucleotide variant in which the G allele replaces the wild‐type C allele, affecting TLR7 mRNA expression. The C allele facilitates binding of the regulatory microRNA miR‐3148, promoting TLR7 mRNA degradation and helping to prevent excessive inflammation and severe disease. In contrast, the G allele impairs miR‐3148 binding, resulting in higher TLR7 mRNA levels [27]. The G allele in rs3853839, unlike the T allele in rs179008, probably causes more and excessive expression of TLR7 and subsequently type I IFN, and possibly excessive inflammation and intensification of COVID‐19, along with adverse effects such as lymphopenia. Excessive type I IFN leads to depletion of CD4+ and CD8+ T cells through induction of apoptosis [59]. In addition, activation of T cells by type I IFN decreases Adenosine triphosphate (ATP) in these cells and thus leads to their death [60]. Another consequence of excessive activation of TLR7, type I IFNs, and ISGs in COVID‐19 is cytokine storm syndrome, which plays an important role in the development of severe disease and can cause the suppression of an effective immune response [59, 61]. According to several studies, excessive inflammation is a major factor in the severity of COVID‐19 [62]. A balanced type I IFN response and controlled inflammation lead to mild disease, whereas overactivation causes immune dysfunction, lung injury, and severe pneumonia [16, 21, 62, 63, 64]. In individuals with the CC genotype, type I IFN is optimally produced, and TLR7 mRNA can be degraded when unnecessary, preventing excessive inflammation and supporting recovery. Conversely, the G allele disrupts miRNA regulation, leading to elevated TLR7 mRNA, overproduction of type I IFN, and ISG overactivation, contributing to severe inflammation [27, 52, 55, 65]. The significant pooled allele analysis further corroborates this risk direction by demonstrating a clear overall G allele burden in severe cases (OR = 1.49, *p* = 0.043), reinforcing the functional relevance of this variant across both sexes.

We found no significant association between the SNP genotypes and any clinical parameter among severe patients.

## 5 Conclusion

As TLR7 is essential for type I IF production, these SNPs likely influence COVID‐19 pathogenesis by modulating TLR7 expression. Our findings suggest that the rs179008 T allele, which may reduce TLR7 expression, is associated with milder disease, whereas the rs3853839 G allele, which impairs miR‐3148‐mediated degradation of TLR7 mRNA, is associated with severe COVID‐19. Taken together, these findings suggest that high TLR7 expression may drive excessive inflammation, while rs179008 confers protection and rs3853839 contributes to disease severity in COVID‐19. Further studies in larger cohorts, including direct measurement of TLR7 and IFN‐I expression, are needed to clarify their roles and inform COVID‐19 treatment and vaccine strategies.

## Author Contributions

Maryam Noroozi participated in conceptualization, performed DNA extraction, PCR‐RFLP, and data analysis, and wrote the manuscript. Gholamreza Daryabor participated in conceptualization, data analysis, and drafting the manuscript. Reza Shafiei and Mona Fani contributed to data analysis, writing, and manuscript revision. Seppo Meri participated in conceptualization, data analysis, and critically revised the manuscript. Mona Fani and Kurosh Kalantar supervised the research and participated in conceptualization, data analysis, writing, and critical revision of the manuscript.

## Ethics Statement

The study received approval from the Shiraz University of Medical Sciences ethics committee (ethical code: IR.SUMS.SEC.1401.569) and the North Khorasan University of Medical Sciences ethics committee (ethical code: IR.NKUMS.REC.1402.035).

## Consent

Informed consent was obtained from participants before their inclusion in the study.

## Conflicts of Interest

The authors declare no conflicts of interest.

## Data Availability

The authors have nothing to report.
